# Clinical and Prognostic Pan-Cancer Analysis of N6-Methyladenosine Regulators in Two Types of Hematological Malignancies: A Retrospective Study Based on TCGA and GTEx Databases

**DOI:** 10.3389/fonc.2021.623170

**Published:** 2021-03-18

**Authors:** Xiangsheng Zhang, Liye Zhong, Zhilin Zou, Guosheng Liang, Zhenye Tang, Kai Li, Shuzhen Tan, Yongmei Huang, Xiao Zhu

**Affiliations:** ^1^ The Marine Biomedical Research Institute of Guangdong Zhanjiang, Guangdong Medical University, Zhanjiang, China; ^2^ Department of Hematology, Guangdong Provincial People’s Hospital, Guangdong Academy of Medical Sciences, Guangzhou, China; ^3^ The Marine Biomedical Research Institute of Guangdong Zhanjiang, Zhanjiang, China; ^4^ Southern Marine Science and Engineering Guangdong Laboratory (Zhanjiang), Zhanjiang, China; ^5^ The Key Lab of Zhanjiang for R&D Marine Microbial Resources in the Beibu Gulf Rim, Guangdong Medical University, Zhanjiang, China

**Keywords:** hematological malignancies, m6A methylation regulators, pan-cancer analysis, prognosis, risk scores

## Abstract

N6-methyladenosine (m6A) is one of the most active modification factors of mRNA, which is closely related to cell proliferation, differentiation, and tumor development. Here, we explored the relationship between the pathogenesis of hematological malignancies and the clinicopathologic parameters. The datasets of hematological malignancies and controls were obtained from the TCGA [AML (n = 200), DLBCL (n = 48)] and GTEx [whole blood (n = 337), blood vascular artery (n = 606)]. We analyzed the m6A factor expression differences in normal tissue and tumor tissue and their correlations, clustered the express obvious clinical tumor subtypes, determined the tumor risk score, established Cox regression model, performed univariate and multivariate analysis on all datasets. We found that the AML patients with high expression of IGF2BP3, ALKBH5, and IGF2BP2 had poor survival, while the DLBCL patients with high expression of METTL14 had poor survival. In addition, “Total” datasets analysis revealed that IGF2BP1, ALKBH5, IGF2BP2, RBM15, METTL3, and ZNF217 were potential oncogenes for hematologic system tumors. Collectively, the expressions of some m6A regulators are closely related to the occurrence and development of hematologic system tumors, and the intervention of specific regulatory factors may lead to a breakthrough in the treatment in the future.

## Background

Hematological malignancies are generally diseases, especially AML and DLBCL. Recent studies have shown that AML is the result of abnormal proliferation and differentiation of myeloid leukocytes. It was found that MELLT3 and METTL14 play a cancer-promoting role in AML, promoting the translation of MYC, MYB, BCL2, SP1, and PTEN, thereby leading to significant gene mutations and causing great difficulties in treatment. Therefore, the introduction of m6A methylation regulator into hematological malignancies is a worthy direction to be explored. N6-methyladenosine (m6A) is the most abundant form of eukaryotic mRNA modification (1, 2), which occurs in the sixth nitrogen atom (N) of adenine (A). As is known to all, it plays a crucial role in the regulation of gene expression, protein transformation, and other biological activities by affecting the stability, translation efficiency, variable splicing, and localization of mRNA (2, 3).

Studies found that m6A methylation requires three types of molecular involvement: writers, erasers, and readers (4, 5). Writers are a methyltransferase complex that writes methylation modification into mRNA and mediates methylation modification of RNA (6). The major molecules involved are METTL3, METTL14, METTL16, KIAA1429, WTAP, ZNF217, RBMX, RBM15, CBLL1, and ZC3H13 (7–12). Erasers are a demethylase complex that removes methyl groups from mRNA and mediates the demethylation of RNA. The major molecules involved are FTO and ALKBH5 (13–15). Readers (16) are m6A-binding proteins to recognize the information of RNA methylation and participate in the transformation and degradation of RNA to produce different functions. The molecules involved are mainly YTHDC1, YTHDC2, YTHDF1, YTHDF2, YTHDF3, HNRNPA2B1, HNRNPC, IGF2BP1, IGF2BP2, and IGF2BP3 (17, 18). The three processes of m6A writers, erasers, and readers are dynamically reversible. They interact with each other to jointly regulate the functions of various gene expressions, leading to changes in cell proliferation (19), apoptosis (20), and self-renewal ability (21), thus affecting the occurrence and development of the disease.

With the in-depth discovery of research, m6A methylation regulators play an important role in the occurrence and development of diseases, especially in cancer (22, 23). For example, Zhu et al. (24) found that ALKBH5 was closely related to the poor prognosis of Non-Small Cell Lung Cancer (NSCLC), and ALKBH5 affected the progression of NSCLC by inhibiting the stability and protein synthesis of TIMP3. Therefore, we obtained the datasets of AML and DLBCL datasets from TCGA, systematically analyzed the 23 widely reported m6A methylation regulators expression differences between normal and cancerous tissue, assessed the m6A regulation of tumor-associated genes and discovered the relationship of hematological malignancies between the clinically relevant factors. All in all, through this study, it is possible to further reveal the mechanism of the development of hematological malignancies, and provide references for future research and clinical treatment.

## Methods

### Data Acquisition

RNA-seq transcriptome data and the corresponding clinicopathological and prognostic information were obtained from TCGA_AML (acute myeloid leukemia, n = 200), TCGA_DLBCL (diffuse large b-cell lymphoma, n = 48). The normal control datasets of AML were obtained from GTEx_Whole Blood (n = 337) and the normal control datasets of DLBCL were obtained from GTEx_Blood Vessel Artery (Aorta, Coronary and Tibial) (n = 606) (**Table 1**). To comprehensively analyze the common characteristics of m6A methylation regulators in the occurrence and development of tumors in the blood system, we summarized the data of AML and DLBCL (normal count 943, tumor count 248) as the “Total” datasets. The expression profiles data were derived from the standardized RNA-seq data of TCGA and GTEx, and the batch effect was removed through the “sva” package to eliminate the influence between the datasets.

**Table 1:** RNA-seq data set of tumor group and control group.

Cancer types	Tumor count	Normal count	Amount
AML	TCGA_AML 200	GTEx_Whole Blood 337	537
DLBCL	TCGA_DLBCL 48	GTEx_Blood vascular Artery 606	654
Total	TCGA_AML 200+ TCGA_DLBCL 48 = 248	GTEx_Whole Blood 337+ GTEx_Blood vascular Artery606 = 943	1191

AML, acute myeloid leukemia; DLBLC, diffuse large b-cell lymphoma; total, AML + DLBLC.

### Selection of m6A RNA Methylation Regulators

We collected 23 widely reported m6A RNA methylation regulators (2, 25–29), studied their expression differences in normal and cancerous tissues, and evaluated the relationship between m6A regulation of tumor-related genes and clinically relevant factors in the hematological malignancies.

### Bioinformatics and Statistical Analysis

**Figure 1:**
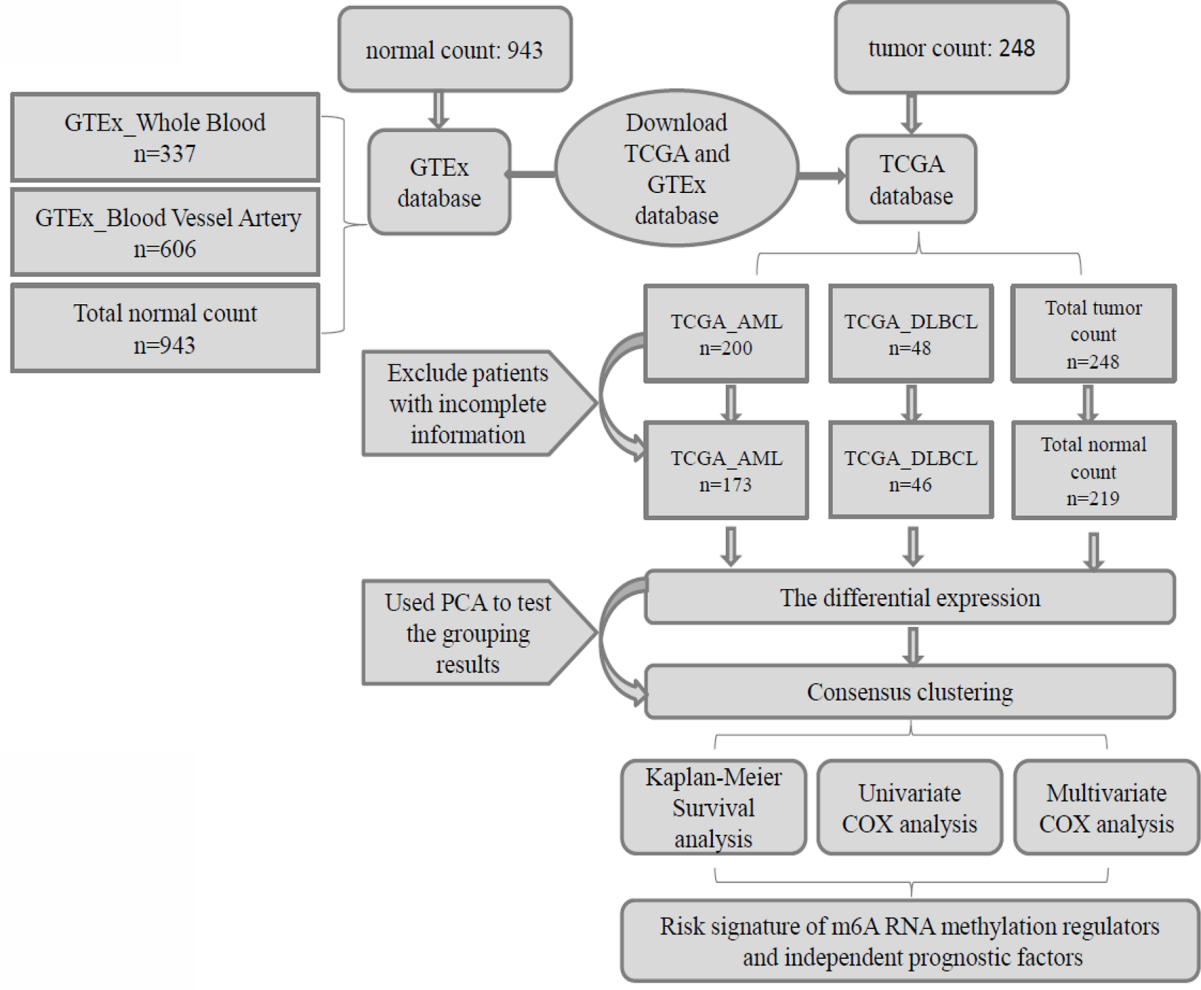
Clinical data and research process of the analysis of pan-carcinoma of m6A RNA methylation regulators in hematological malignancies.

In this study, we used Practical Extraction and Report Language (Perl) quickly and accurately processes the file paths that require R package analysis, then we used the R package (R v3.5.2 and R v3.6.2) for data analysis. Firstly, the “limma” package was used to analyze the differential expression of 23 m6A RNA methylation regulators in patients and normal control group, and the “pheatmap” package and “vioplot” package were used to visualize the differential expression of 23 regulators, and then the “corrplot” package was used to analyze the correlation of tumor gene expression. Secondly, we used “consensus cluster plus” package to cluster and group the patient data in AML, DLBCL, and “Total” into “cluster 1” subgroups and “cluster 2” subgroups, and used “gmodels” package to visualize the grouping information. Thirdly, we deleted the incomplete clinical information, used the “survival” package to conduct grouping survival analysis and grouping clinical correlation tests, and used the “pheatmap” package to visualize the expression differences of the 23 regulators between “cluster 1” subgroups and “cluster 2” subgroups. Fourthly, we used “forestplot” package and “survival” package COX proportional single variable regression analysis, and used “glmnet” package to filter out meaningful m6A methylation regulatory factor, and used “survival” package and “survival ROC” package to construct Lasso regression model to analyze the survival situation, and used “pheatmap” package to visualize the related regulatory factor expression and the relationship between the different clinical factors. Finally, we used “forestplot” package and “survival” package to conduct independent single-factor and multi-factor prognostic analysis and screened out the clinical factors affecting the prognosis (**Figure 1**).

To better understand the prognostic role of m6A RNA methylation regulators in patients with hematological malignancies, we use the Cox univariate analysis data from the TCGA and GTEx database to select the regulators significantly related to survival (P < 0.05), and developed a potential risk signature using LASSO Cox regression algorithm. Risk score calculation is as follows:

$$\text{Risk score}=\sum_{i=1}^{n}\text{Coe}{\text{f}}_{i}\times {\text{x}}_{i}$$

Coef*_i_* is the coefficient, and x*_i_* is the expression value of each selected gene (30–34). This formula was used to calculate the risk scores.

## Results

### Regulations of m6A RNA Methylation in Hematological Malignancies

**Figure 2:**
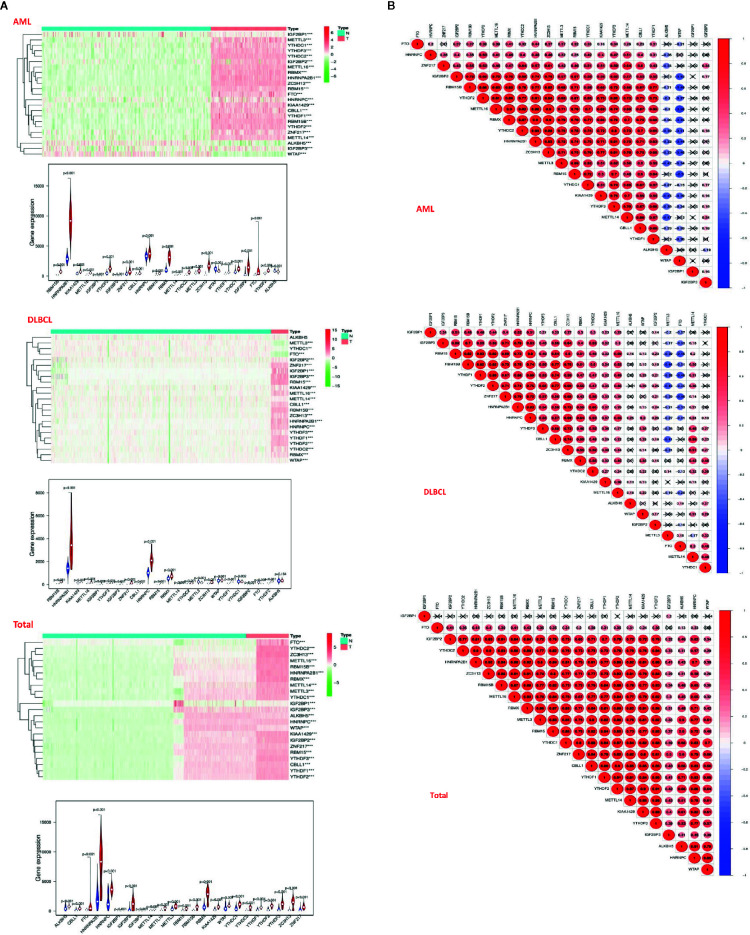
Distribution of m6a RNA methylation regulators in hematological malignancies. **(A)** The “heatmaps” shows the expression levels of 23 m6A RNA methylation regulators in hematological malignancies. The higher expression, the darker the color (red for up-regulated, green for down-regulated), and the tree diagram above represents the clustering results of different samples from different experimental groups, while the tree on the left shows the clustering analysis results of different regulators from different samples; The “vioplot” visualized the differential m6A regulators (assume blue is normal tissue and red is tumor tissue). **(B)** The “corrplot” shows the correlation analysis of the expression of 23 m6A regulators in hematological malignancies.

Due to the crucial role of m6A methylation regulators in tumorigenesis and development, we first used “limma” package to analyze the expression levels of 23 m6A RNA methylation regulators in 200 AML patients in TCGA datasets with 337 normal controls in GTEx datasets, and 48 DLBC patients in TCGA datasets with 606 normal controls in GTEx datasets. Then, the datasets of AML and DLBCL were summarized as the “Total” datasets (normal count 943, tumor count 248) was used to comprehensively analyze the role of m6A methylation regulators in tumor development. And then we used “pheatmap” package and “vioplot” to visualize the results of the differential expression of the 23 regulators. The results showed that AML patients commonly contain a higher proportion of ALKBH5, CBLL1, FTO, HNRNPA2B1, HNRNPC, IGF2BP1, IGF2BP2, IGF2BP3, METTL14, METTL16, METTL3, RBM15, RBM15B, RBMX, KIAA1429, WTAP, YTHDC1, YTHDC2, YTHDF1, YTHDF2, YTHDF3, ZC3H13, ZNF217 (**Figure 2A**). DLBC patients commonly contain a higher proportion of RBM15, HNRNPA2B1, KIAA1429, METTL16, IGF2BP1, YTHDF3, IGF2BP3, ZNF217, CBLL1, HNRNPC, RBM15, RBMX, METTL14, YTHDC2, METTL3, ZC3H13, WTAP, YTHDF1, YTHDC1, IGF2BP2, FTO, YTHDF2 (**Figure 2B**). Then, the analysis of the “Total” data of AML and DLBC revealed that the proportion of 23 m6A RNA methylation regulators was significantly higher than that of the normal control group (**Figure 2A**).

Next, we used “corrplot” package to analyze the correlation between m6A RNA methylation regulators in different datasets. It was found that the correlation of different m6A RNA methylation regulators was from weak to strong. The highest correlation between HNRNPA2B1 and ZC3H13 was found in the AML datasets (**Figure 2A**). The correlation between YTHDF1 gene and YTHDF2 gene was the strongest in the DLBC datasets (**Figure 2B**). In the “Total” datasets, YTHDF1 gene and YTHDF2 gene are the most correlated (**Figure 2B**). These results indicated that the differential expression of m6A RNA methylation regulators was closely related to hematological malignancies.

### Consensus Clustering of m6A RNA Methylation Regulators Identified Two Clusters of Hematological Malignancies

**Figure 3:**
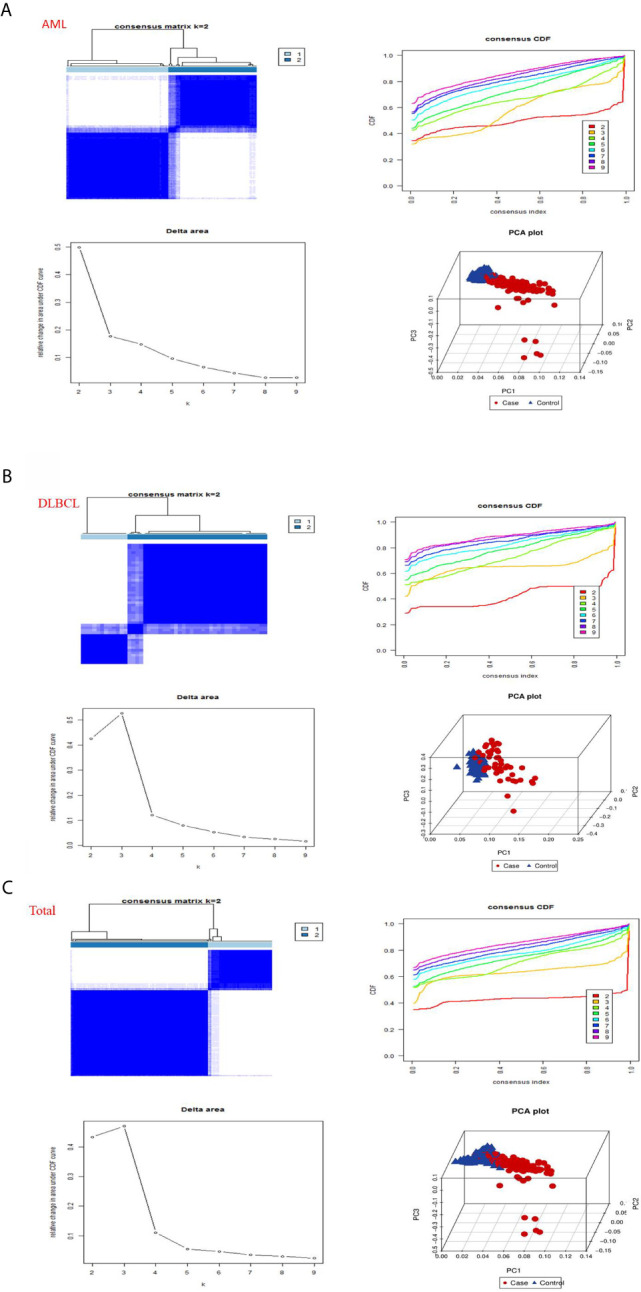
Consistent clustering by m6a RNA methylated modulators in hematological malignancies. **(A)** Identification of consistent clustering by m6a RNA methylated modulators in AML datasets. Is the consistency clustering matrix of k = 2, and the cumulative distribution function of consistency clustering (CDF) when k = 2–9, and the relative change of the area under the CDF curve when k = 2–9 and the 3D principal component analysis (3D PCA) of total TCGA RNA expression profile datasets and the “cluster 1”subtype is marked in red and the “cluster 2”subtype is marked in blue. **(B)** Identification of consistent clustering by m6a RNA methylated modulators in DLBCL datasets. **(C)** Identification of consistent clustering by m6a RNA methylated modulators in “Total” datasets.

Then, we used the “consensus cluster plus” package to cluster the expression correlations of m6A RNA methylation regulators, and the clustering stability increased from k = 2 to 10. In AML, we found that when datasets k = 2, the correlation between the two datasets aggregated was very strong (**Figure 3A**). So we divided the two datasets into two categories: “cluster 1” subgroup and “cluster 2” subgroup (**Figure 3A**). To prevent data contingency, 3d PCA was used to analyze the two datasets, and the results showed that there were significant differences between them and that “cluster 1” subgroup can gather together and “cluster 2” subgroup can also gather together (**Figure 3A**). Therefore, we analyzed the datasets of DLBC and “Total” successively utilizing CCP and PCA. In DLBCL, “cluster 1” subgroup and “cluster 2” subgroup with obvious differences can be aggregated when datasets k = 2 (**Figure 3B**). Moreover, in 3d PCA analysis, results show “cluster 1” subgroup can gather together and “cluster 2” subgroup can also gather together (**Figure 3B**). Similarly, in “Total”, “cluster 1” subgroup and “cluster 2” subgroup with obvious differences can be aggregated when the datasets k = 2 (**Figure 3C**). And, in 3d PCA analysis, the results show that “cluster 1” subgroup can gather together and “cluster 2” subgroup can also gather together (**Figure 3C**). These results manifested that the m6A RNA methylation regulator we have classified is reliable.

### Categories Identified by Consensus Clustering Are Associated With Clinical Outcomes and Clinicopathological Characteristics

**Figure 4:**
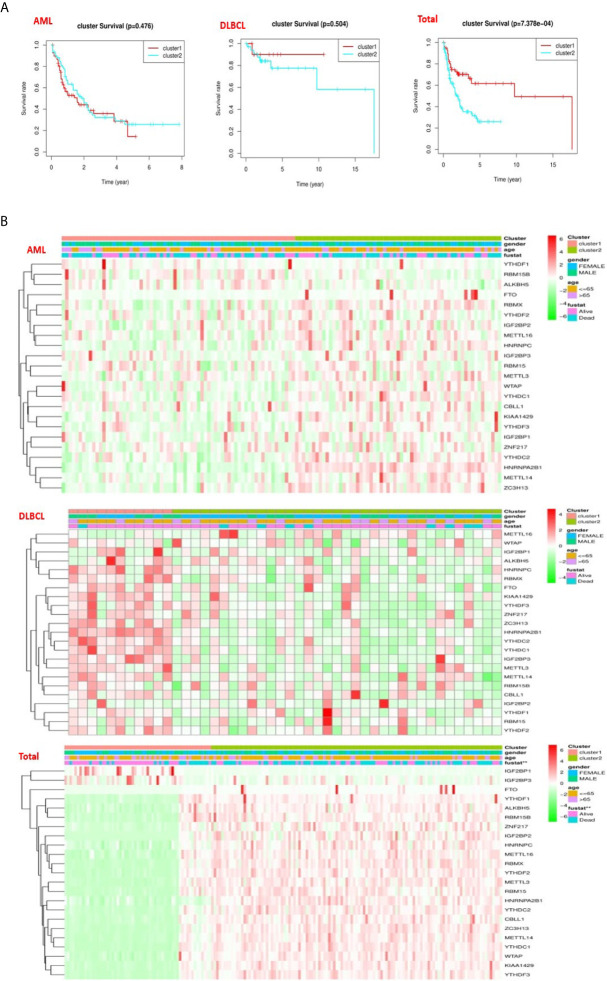
Differences in clinicopathologic features and overall survival of hematological malignancies. **(A)** The “Kaplan–Meier” overall survival (OS) curve of hematological malignancies in two clusters (cluster 1/2) defined by consistent expression of m6a RNA methylation regulators in hematological malignancies. **(B)** The “heatmap” and clinicopathologic features of two clusters defined by consistent expression of the m6A regulatory genes (clusters1/2).

To better comprehend the clustering results, clinical results, and clinicopathological characteristics, we first deleted the incomplete clinical information, and then used the “survival” package to conduct grouping survival analysis and grouping clinical correlation tests, and used the “pheatmap” package to visualize the expression differences of the 23 regulators between “cluster 1” subgroup and “cluster 2” subgroup. We found that in AML (P = 0.476) and DLBC (p = 0.505), there was no significant difference in overall survival rate (OS) between the “cluster 1” subgroup and the “cluster 2” subgroup (**Figure 4A**), which may lack sufficient data and may require longer follow-up observation of such patients. However, we found in “Total” that there was a significant difference in OS between “cluster 1” subgroup and “cluster 2” subgroup (**Figure 4A**), and survival rate of “cluster 2” subgroup was significantly shorter than that of “cluster 1” subgroup, and most m6A RNA methylated modulators were highly expressed in “cluster 2” subgroup (**Figure 4B**). By comparing the subgroup of “cluster 1” and “cluster 2” in AML, DLBCL, and “Total”, it is found that “cluster 2” subgroup are closely related to the survival state (**Figure 4A**). Therefore, we found that the clustering results were closely related to the degree of malignancy of the tumor.

### Prognostic Value of Risk Signature and m6A RNA Methylation Regulators

**Figure 5:**
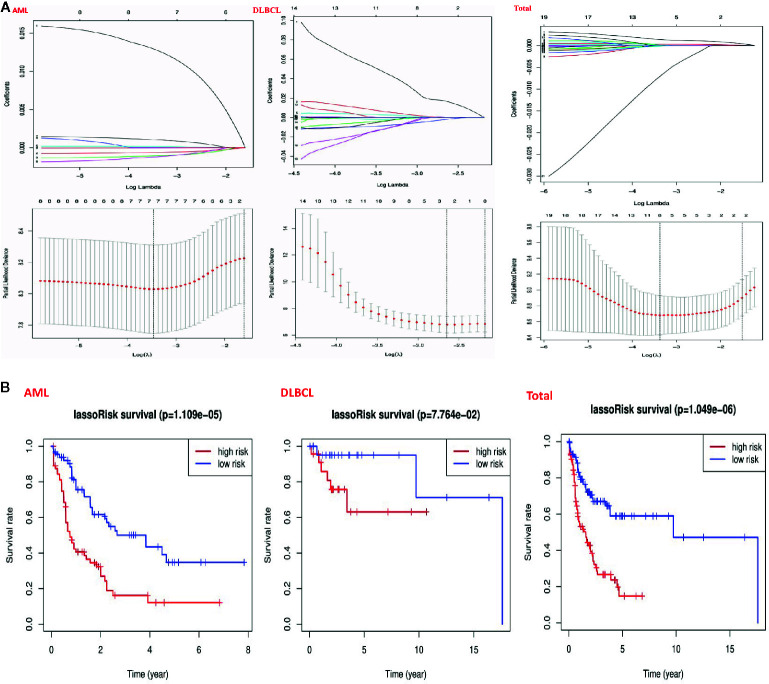
Risk signatures with m6A RNA methylation regulators in hematological malignancies. **(A)** The process of building the signature containing 23 m6A RNA methylation regulators and used “glmnet” to filter out meaningful m6A methylation regulatory factor in AML, DLBCL and “Total” datasets; **(B)** The coefficients calculated by multivariate Cox regression using LASSO are shown and “Kaplan–Meier” overall survival (OS) curves for patients in the TCGA datasets assigned to high and low-risk groups according to the risk score.

In order to understand the prognostic role of m6A RNA methylation regulators in hematological malignancies, we performed a univariate Cox regression analysis on the expression levels in the TCGA datasets. Analysis of AML datasets revealed that high expression of IGF2BP3, IGF2BP2, and ALKBH5 have a worse survival in AML patients (**Figure 5A**). On the contrary, high expression of YTHDF3, YTHDC2, CBLL1, and HNRNPA2B1, have a better survival in AML patients (**Figure 5A**). Analysis of DLBCL datasets shows that high expression of METTL14 has a worse survival in DLBC patients (**Figure 5A**). On the contrary, high expression of IGF2BP1 has better survival in DLBC patients (**Figure 5A**). Finally, Analysis of “Total” datasets revealed that ALKBH5, IGF2BP2, RBM15, METTL3, ZNF217 have a worse survival (**Figure 5A**). On the contrary, high expression of YTHDF1, YTHDF3, and IGF2BP1 have a better survival (**Figure 5A**).

Then, we used the “survival” package and the “survival ROC” package to analyze 23 prognostic genes in the TCGA datasets by applying the least absolute shrinkage and selection operator (LASSO) Cox regression algorithm. Based on the least criteria, risk characteristics were established and genes closely related to the prognosis of hematologic malignancies were selected. Finally, the data obtained from LASSO algorithm can be divided into low-risk group and high-risk group according to the median risk score. In the AML datasets, based on the least criteria, we obtained 7 genes closely related to AML: IGF2BP3, ALKBH5, IGF2BP2, YTHDF3, YTHDC2, CBLL1, and HNRNPA2B1. According to the data obtained in LASSO algorithm, we divided them into the low-risk group and high-risk group according to the median risk score (**Figure 5B**). By studying the risk prognosis of these seven genes, it was found that the survival time was shorter in the high-risk group (**Figure 5B**). In the datasets DLBCL, we obtained two genes closely related to DLBCL: METTL14 and IGF2BP1. According to the data obtained in LASSO algorithm, we divided them into the low-risk group and high-risk group according to the median risk score (**Figure 5B**). By studying the risk prognosis of these two genes, it was found that the survival period of the high-risk group was shorter (**Figure 5B**). In the “Total” datasets, 8 genes closely related to hematopoietic malignancies were obtained based on the least criteria: ALKBH5, IGF2BP2, RBM15, METTL3, ZNF217, YTHDF1, YTHDF3, and IGF2BP1. According to the data obtained in LASSO algorithm, we divided them into the low-risk group and high-risk group according to the median risk score (**Figure 5B**). By studying the risk prognosis of these 8 genes, it was found that the survival period of the high-risk group was shorter (**Figure 5B**). Therefore, through the above studies, we found that specific m6A regulatory factors are closely related to the development of circulatory system malignant tumors.

### Prognostic Risk Score Is Closely Strong Associations With the Clinical Characteristics of Hematological Malignancies

**Figure 6:**
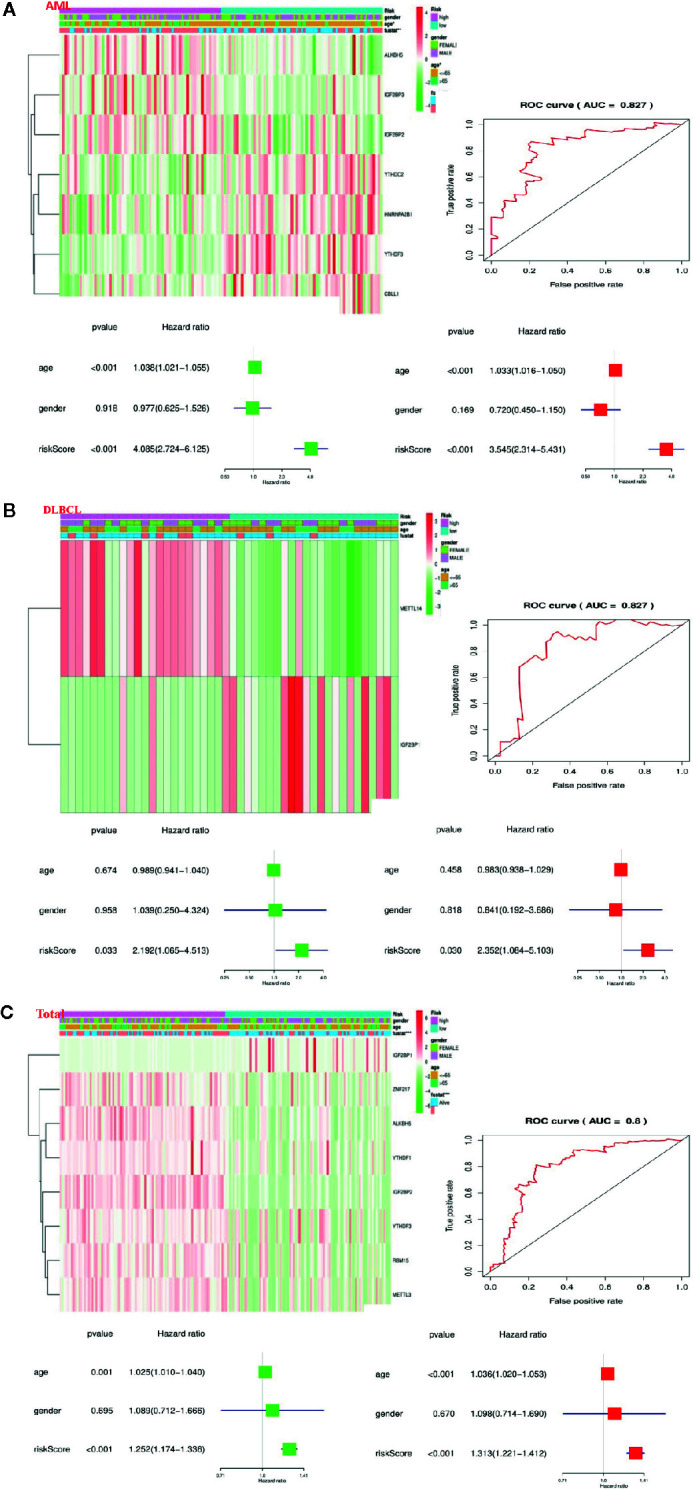
Relationship between the risk score, clinicopathological features, and clusters subgroups in hematological malignancies. **(A)** Relationship between the risk score, clinicopathological features, and clusters subgroups in AML datasets. The “heatmap” shows the expression levels of the m6A RNA methylation regulators in low-risk and high-risk. The distribution of clinicopathological features was compared between the low- and high-risk groups; The ROC curve indicates the predictive efficiency of risk signatures.** **The Univariate Cox regression analyses of the correlation between clinicopathological factors (including the risk score) and overall survival of patients in the TCGA datasets, and the multivariate Cox regression analyses of the relationship between clinicopathological factors (including the risk score) and overall survival of patients in the TCGA datasets. **(B)** Relationship between the risk score, clinicopathological features, and clusters subgroups in DLBCL datasets. **(C)** Relationship between the risk score, clinicopathological features, and clusters subgroups in “Total” datasets.

To better comprehend the clinical results of hematological malignancies in the high-risk group, we thoroughly analyzed the relationships between m6A RNA methylation regulators in the high-risk group and low-risk group patients in the TCGA datasets and the pathological features of hematological malignancies, including gender, age, and survival-state, and found a specific relationship between them. Besides, Patients with AML in the high-risk group generally contain higher proportions of IGF2BP3, ALKBH5, IGF2BP2, YTHDF3, YTHDC2, CBLL1, HNRNPA2B1 (**Figure 6A**). Patients with DLBCL in the high-risk group generally had a higher proportion of METTL14, IGF2BP1 (**Figure 6B**). And in the “Total” data set, it contains a high percentage of ALKBH5, IGF2BP2, RBM15, METTL3, ZNF217, YTHDF1, YTHDF3, IGF2BP1 (**Figure 6C**).

Then, we performed a ROC curve to predict the 5-year survival rate of patients with circulatory system malignant diseases, and the results showed AML (AUC = 0.837) (**Figure 6A**), DLBC (AUC = 0.827) (**Figure 6B**), and “Total” (AUC = 0.800) (**Figure 6C**). Next, we made univariate and multivariate Cox regression analyses for the TCGA dataset to confirm whether the risk signature is an independent prognostic indicator. Both the univariate and multivariate Cox regression analyses in the AML dataset showed that age (P < 0.001) and risk-score (P < 0.001) were correlated with OS (**Figure 6A**). Both the univariate and multivariate Cox regression analyses in the DLBC dataset showed that risk-score (P < 0.005) was related to OS (**Figure 6B**). Both the univariate and multivariate Cox regression analyses in the “Total” dataset showed that age (P ≤ 0.001) and risk-score (P < 0.001) were correlated with OS (**Figure 6C**). Based on the evidence we found, prognostic risk score and age are closely related to the clinicopathological features of hematological malignancies.

## Discussion

Hematological malignancies are listed as one of the top ten domestic high incidence tumors, which is similar to other Asian countries, but significantly lower than European and American countries. Among the hematological malignancies, AML is the most common leukemia (35, 36). And DLBCL is the leading non-hodgkin’s lymphoma (NHL) in the world (37). Moreover, the incidence of these two kinds of hematological malignancies is increasing year by year, which undoubtedly poses a serious threat to human health and life safety. The occurrence and development of hematological malignancies are very rapid and complex, which is a complex process of the interaction between internal genetic factors (38–44), molecular signal transduction (45, 46), metabolic factors (47–49), immune factors (50–55), and tumor microenvironment (41, 56–59). Although current chemotherapy can prolong the survival time of tumor patients (60, 61), the prognosis and economic burden are still difficult problems in clinical work. Therefore, it is urgent to explore the pathogenesis and new therapeutic methods of hematologic malignancy, such as targeted therapy (62–64).

With the in-depth study of m6A RNA methylation regulators, the m6A RNA methylation regulatory factors (65) are closely related to the occurrence and development of malignant tumors (66). Li et al. (1) systematically analyzed m6A methylation levels in 33 types of cancer patients and found that the m6A methylation regulator has a wide range of gene mutations, and identified an m6A methylation regulator closely associated with a variety of cancers: IGF2BP3. Many studies have suggested that m6A RNA methylation regulators primarily regulate tumor cell growth (inhibit or promote) (67). For example, Wang et al. (68) found that the expression level of RHPN1-AS1 of epithelial ovarian cancer (EOC) tissue was significantly higher than that in para cancer tissues, and the expression level of RHPN1-AS1 was closely related to the prognosis. In EOC, m6A methylation regulator increased the stability of the RHPN1-AS1 methylated transcript by regulating the degradation of RNA, up-regulated the expression of RHPN1-AS1, and thereby promoted the occurrence and metastasis of EOC. The discovery of this key gene is promising to treat EOC with a drug target.

However, there are still a few basic studies on the role and biological role of m6A RNA methylation regulators in Hematological Malignancies. In this study, we first analyzed the expression relationship of m6A RNA methylation regulators in AML, DLBCL, and “Total” datasets and normal tissues, and found that the proportion of these 23 m6A RNA methylation regulators was higher than that of the normal control group. This fits our guess. Then, through consistency clustering, we divided two closely related “cluster 1” and “cluster 2” based on m6A RNA methylation regulators, and compared with OS we found that there was a certain correlation between m6A RNA methylation regulators and tumor malignancy, so the next step is to look for methylation regulators that are closely related to tumors. We performed univariate Cox regression analysis of the expression levels of m6A RNA methylation regulators in the TCGA data set, and found that AML patients with high expression of IGF2BP3 ALKBH5 and IGF2BP2 had poor survival. The survival of DLBCL patients with high METTL14 expression was poor. From the analysis on the “Total” data set IGF2BP1, ALKBH5, IGF2BP2, RBM15, METTL3, ZNF217 is the potentially carcinogenic gene in hematological malignancies. In addition, the data obtained from the LASSO algorithm was used to calculate the risk score of the TCGA data set, and it was found that patients in the high-risk group had a shorter survival time. The discovery of these potential oncogenes makes it a new therapeutic strategy to treat AML and DLBCL with drug targets. In order to deeply understand the prognostic risk score and the clinical characteristics of hematological malignancies, we used ROC curve to predict the 5-year survival rate of patients with hematological malignancies, and univariate and multivariate Cox regression analysis showed that age and risk score was significantly correlated with OS. Based on this evidence, we believe that the prognostic risk score and age are closely related to the clinicopathological characteristics of malignant tumors in the hematological system.

## Conclusions

In conclusion, we systematically investigated the expression, potential function, and prognostic value of m6A RNA methylation regulators in hematologic tumors. We found that hematologic tumors are closely related to m6A methylation regulators, which affect the occurrence, development, and clinical prognosis of tumors. Through the study of these closely related methylation regulators provides a direction for the clinical research of targeted therapy.

## Data Availability Statement

The original contributions presented in the study are included in the article/supplementary material. Further inquiries can be directed to the corresponding authors.

## Ethics Statement

The studies involving human participants were reviewed and approved by Guangdong Medical University Ethics committee. Written informed consent for participation was not required for this study in accordance with the national legislation and the institutional requirements.

## Author Contributions

XZ and YH designed and supervised the research. XSZ conducted the bioinformatic and statistical analysis and wrote the manuscript. LZ interpreted the data, edited and discussed the manuscript. ZZ, GL, ZT, KL, and ST assisted bioinformatic and statistical analysis. XZ checked the bioinformatic and statistical accuracy as an expert. All authors contributed to the article and approved the submitted version.

## Funding

This work was supported partly by National Natural Science Foundation of China (81541153), Guangdong Science and Technology Department (2016A050503046, 2015A050502048, and 2019B090905011), The Fund of Southern Marine Science and Engineering Guangdong Laboratory (Zhanjiang) (ZJW-2019-007), The Public Service Platform of South China Sea for R&D Marine Biomedicine Resources (GDMUK201808), The Science and Technology Program of Zhanjiang (2017A06012), The Guangdong University Youth Innovation Talent Project (2020KQNCX023), The Scientific Research Fund of Guangdong Medical University (GDMUM201819 and GDMUM202002), and non-funded science and technology project of Zhanjiang City (2020B01007). The funders had no role in the design of the study, the collection, analysis, and interpretation of the data, the writing of the manuscript, and the decision to submit the manuscript for publication.

## Conflict of Interest

The authors declare that the research was conducted in the absence of any commercial or financial relationships that could be construed as a potential conflict of interest.

## Abbreviations

AML, acute myeloid leukemia; DLBLC, diffuse large b-cell lymphoma; TCGA, The Cancer Genome Atlas; GTEx, genotype-tissue expression; NSCLC, non-small-cell lung cancer; CCP, Consensus Cluster Plus; PCA, principal component analysis; Perl, Practical Extraction and Report Language; LASSO, Least Absolute Shrinkage and Selection Operator; NHL, non-Hodgkin’s lymphoma; EOC, epithelial ovarian cancer.
